# Modeling and Control for HIV/AIDS Transmission in China Based on Data from 2004 to 2016

**DOI:** 10.1155/2017/8935314

**Published:** 2017-09-12

**Authors:** Zhiming Li, Zhidong Teng, Hui Miao

**Affiliations:** ^1^College of Mathematics and System Sciences, Xinjiang University, Urumqi, Xinjiang, China; ^2^College of Applied Mathematics, Shanxi University of Finance and Economics, Taiyuan, Shanxi, China

## Abstract

HIV is one of the major life-threatening viruses that are spreading in the People's Republic of China (China for short). A susceptible-exposed in the latent stage-infectious (SEI) model is established to sketch the evolution of epidemic. The basic reproduction number is defined. By constructing Lyapunov function, globally asymptotical stabilities of the disease-free and endemic equilibria are given. Then, optimal control theory is applied in HIV/AIDS epidemic. Precaution, screening, and treatment of control variables are introduced and a new model with control is established. Through the HIV/AIDS data in China, all parameters involved in SEI model are analyzed and parts of them are estimated. Further, by control model, optimal strategy is obtained. Results show that the precaution and treatment are the major contributors to preventing and controlling HIV/AIDS epidemic.

## 1. Introduction

Acquired immunodeficiency syndrome (AIDS) is a serious life-threatening disease, caused by human immunodeficiency virus (HIV). HIV can affect T lymphocytes (CD4+) cells of human immune system and destroy so many of these cells that the body cannot fight off infections and disease, leading to a variety of opportunistic infections and death [1]. The main transmission routes are needle sharing between injecting drug users (IDUs), commercial sex between female sex workers (FSWs) and clients, unsafe blood collection, and maternal-infant vertical transmission [1]. It is rapidly spreading in the world ever since it was firstly detected in 1981 [2]. According to Global Health Observatory (GHO) data of World Health Organization (WHO): at the end of 2013, almost 78 million people have been infected with HIV virus and about 39 million people have died of HIV/AIDS, where about 35.0 million people were living with HIV/AIDS and 1.5 million people died of AIDS-related illnesses worldwide [3]. Now, HIV/AIDS is one of the biggest social, economic, and health issues in the world.

China's first AIDS case, a dying tourist, was identified in 1985; see [4, 5]. In 1989, the first indigenous cases were found among heroin users in Yunnan Province near China's southwest border. With increasing market economy and more migrant workers, HIV quickly spreads to major population centers or the regions with high degrees of population migration, including Guangdong, Guangxi, Henan, Sichuan, and Xinjiang Provinces [6]. By 1998, HIV had reached all 31 provinces, autonomous regions, and municipalities [7]. The incidence rate is in a phase of exponential growth. After 2004, the epidemic has concentrated in high-risk groups [8]. More and more people living with HIV began to progress to clinical AIDS. Particularly, the number of HIV/AIDS patients has been increasing quite fast over the last few years in China. More detailed evolution of HIV/AIDS epidemic in China can be found in [8–12].

However, HIV/AIDS is still an incurable disease at this time. There is no treatment or prophylaxis that can cease disease progression, even though existing therapeutic regimes with drug antiretroviral therapy (ART) and methadone maintenance treatment (MMT) are successful in extending life expectancy. In order to gain a quantitative insight into HIV/AIDS transmission dynamics and suggest the effective control strategies, a number of dynamic mathematical models of HIV/AIDS transmission have emerged as important tool; see [13–21]. These dynamic models are based on the nature of disease and the type of population. Denote $\dot{f}(t)=df(t)/dt$. Let *S*(*t*), *E*(*t*), and *I*(*t*) be the numbers of susceptible, exposed in the latent stage, and infected individuals at time *t*, respectively. For HIV/AIDS epidemic, the classical dynamic model is the susceptible-exposed in the latent stage-infectious (SEI) model as follows:

(1) S˙t=Λ−βStIt−μSt,E˙t=βStIt−μ+σEt,I˙t=σEt−μ+μ1It.

In model (1), Λ is a constant recruitment rate of susceptible immigration, 1/*σ* is the mean latent period, *μ* is the natural death, and *μ*_1_ is the case fatality rate per unit time. The incidence term is of the bilinear mass-action form *βSI*, and *β* is the transmission rate per unit time. However, in addition to sexual contact, the transmission of HIV is by direct contact with blood and breast milk from infectious individuals. If one progresses to the exposed in the latent stage of HIV infection, he (or she) has highly infectious, too.

In this paper, we modify the SEI model (1) by assuming that patients in the exposed stage were infectious and apply the control theory to provide optimal strategies, where time dependent control strategies have been applied to the studies of disease transmission [22–25]. The rest of the work is organized as follows. In Section 2, the data of the reported HIV/AIDS and death cases in China are collected from January 2004 to December 2016. In Section 3, we establish a modified SEI model and analyze some properties of the disease-free and endemic equilibria. In Section 4, the precaution, screening and treatment control functions are introduced and a control model is established. Based on the data set, parameter estimation is given in Section 5 and optimal control strategies are provided in Section 6. In Section 7, discussion and conclusion are shown.

## 2. Data of HIV/AIDS and Death Cases in China

The transmission of HIV is by direct contact with blood, semen, preseminal fluid, rectal fluid, vaginal fluid, and breast milk from infectious individuals, which does not spread by air, water, insects, saliva, tears, casual contact, and toilet seats. Unlike some other viruses, the human body cannot get rid of HIV, where once one has HIV, one has it for life. Based on the international classification of diseases (ICD) and ICD-10 code [26, 27], the incidence of HIV infection has a complete process that is divided into three phases: acute infection period (B23.0), incubation period (Z20.6, Z21), and typical of AIDS (B20~B24).

One individual was just infected with HIV; there is exposed process in the latent period. During this period, the patient has infectivity. During the early stage of HIV infection, HIV reproduces at very low levels. Thus, people with HIV have no symptoms, or only mild ones. After HIV infection, some of people start with fever, headache, swollen glands, sore throat, rash, fatigue, muscle, and joint pains. These symptoms can last for a few days to several weeks or months. Some of the people with HIV do not have any symptoms at all for 10 years or more. With ART treatment or proper medical care, HIV can be controlled so that the life expectancy is extended. The time needed for depletion of T lymphocytes population to the point that opportunistic infections and malignancies occur represents the incubation period. If left untreated, HIV can destroy the immune system of the body that cannot fight off infections and disease. Further, one will progress to AIDS, the late stage of HIV infection. Some symptoms include rapid weight loss, recurring fever or profuse night sweats, extreme and unexplained tiredness, pneumonia, and memory loss. Without treatment, people with AIDS typically survive about 3 years. Thus, it is essential that we understand the efficacy of current HIV/AIDS management strategies so that we can develop supplementary measures that will delay or prevent the progression of AIDS and, ultimately, reduce the rate of mortality.

**Figure 1:**
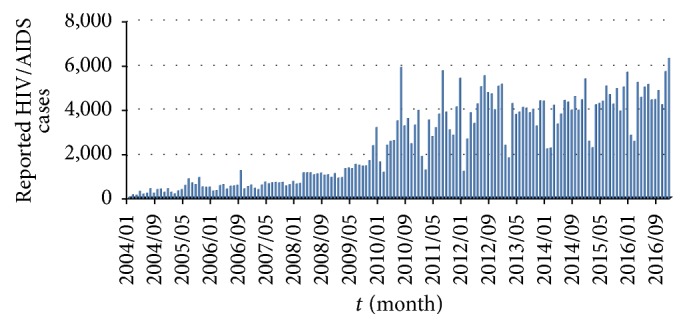
Reported HIV/AIDS individuals by month, 2004–2016.

**Figure 2:**
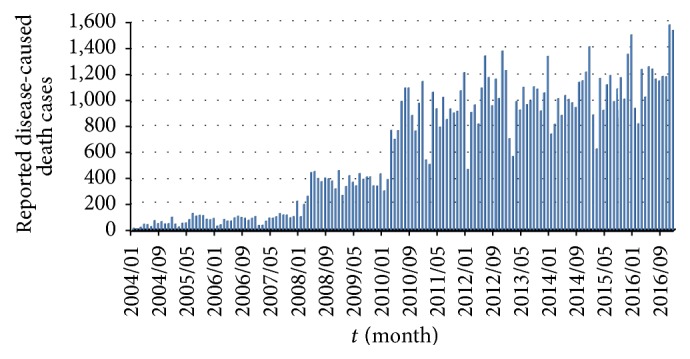
Reported disease-caused death individuals by month, 2004–2016.

The first step is to try to collect publicly available data relevant for the HIV/AIDS epidemic in China. Based on National Health and Family Planning Commission (NHFPC) of the People's Republic of China, the Chinese Center for Disease Control and Prevention (CDC), National Bureau of Statistics (NBS) of the People's Republic of China, and National Bureau of Statistics of China (National data), the data of new reported HIV/AIDS individuals and new disease-caused death cases is obtained from January 2004 to December 2016; see Figures 1 and 2.

**Figure 3:**
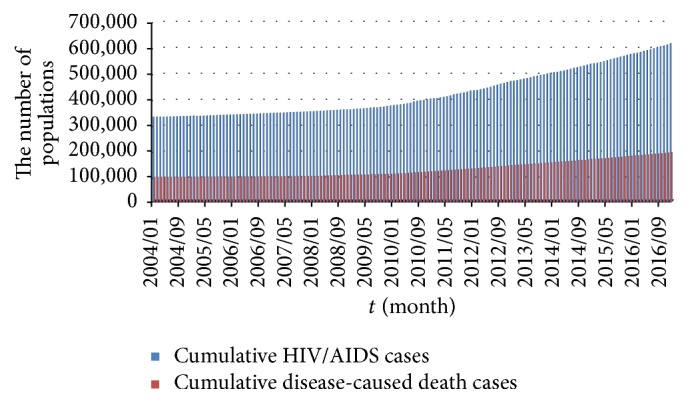
Cumulative HIV/AIDS and disease-caused death cases by month, 2004–2016.

According to CDC, the Chinese Center for Disease Control and Prevention Sexually Transmitted Disease of AIDS Prevention and Control Center (NCAIDS/STD), by August 31, 2015, it has been reported that the alive HIV/AIDS patients had reached 561,807, and the death cases had reached 173,180 [28]. From January 1, 2004, to August 31, 2015, the cumulative alive HIV/AIDS cases are 308,400 and death cases reached 77,384. Thus, by the end of 2003 the cumulative number of alive HIV/AIDS cases is around 330,791 (=561807 − 308400 + 77384) and death cases are 95,796 (=173180 − 77384). Then, cumulative alive HIV/AIDS cases and death cases are 330,839 (=330,791 + 60 − 12) and 95,808 (=95,796 + 12) in January 2004, respectively. From Figures 1 and 2, we obtain the numbers of the cumulative alive HIV/AIDS people and disease-caused cases. For instance, in February 2004, the number of cumulative alive HIV/AIDS cases is 330,839 + 170 − 10 = 330,999. Moreover, from January 2004 to December 2016, the cumulative number of disease-caused death cases equals the sum of death cases in last month and new cases of this month; see Figure 3. Through the cumulative number of HIV/AIDS epidemic in China, we investigate HIV/AIDS transmission dynamics and provide the effective control strategies.

## 3. SEI Epidemic Model

Suppose the total population at time *t*, denoted by *N*(*t*), is divided into three mutually exclusive classes: susceptible individuals *S*(*t*) and undiagnosed individuals *E*(*t*) in the latent period and infected individuals *I*(*t*) who have been diagnosed. Hence, *N*(*t*) = *S*(*t*) + *E*(*t*) + *I*(*t*). After one unit time, the susceptible population is generated by recruitment of individuals at a rate Λ. The susceptible individuals may be reduced by the effective contact with HIV infected individuals at the rate (*β*_1_*E*(*t*) + *β*_2_*I*(*t*)). Further, it may be decreased by natural death rate *μ*. Among HIV/AIDS patients, some people with HIV have not be found or diagnosed who may be unaware of their infection. The greater number of susceptible people is at risk of infection. Thus, the population of class *E*(*t*) at time *t* is increased by the infection of susceptible individuals at (*β*_1_*E*(*t*) + *β*_2_*I*(*t*)). Moreover, it may be reduced by the infection diagnosed at rate *σ* as well as the natural death rate *μ*. The population of class *I*(*t*) is increased by the diagnosed HIV individuals who come from class *E*(*t*). And it can be decreased through natural death as well as case fatality rate *μ*_1_.

Based on these classes *S*(*t*), *E*(*t*), and *I*(*t*), the model for transmission dynamics of HIV/AIDS is given by the following ordinary differential equations:

(2) S˙t=Λ−β1StEt−β2StIt−μSt,E˙t=β1StEt+β2StIt−μ+σEt,I˙t=σEt−μ+μ1It

with initial conditions *S*(0) = *S*_0_ > 0, *E*(0) = *E*_0_ > 0, and *I*(0) = *I*_0_ ≥ 0.

Through fundamental theory of functional differential equation [29], model (2) admits a unique solution (*S*(*t*), *E*(*t*), *I*(*t*)) satisfying initial conditions. It is easy to know that solution of model (2) with initial conditions is defined on [0, +*∞*) and remains positive for all *t* ≥ 0.

**Theorem 1 .**

Let (*S*(*t*), *E*(*t*), *I*(*t*)) be solution of model (2) satisfying initial conditions. Then, *S*(*t*), *E*(*t*), and *I*(*t*) are ultimately bounded.

In fact, by positivity of solution of model (2), we have

(3) N˙t=Λ−μSt+Et+It−μ1It≤Λ−μNt.

Therefore, for all sufficiently large *t* we have *N*(*t*) ≤ Λ/*μ*. This implies that *S*(*t*), *E*(*t*), and *I*(*t*) are bounded. For model (2), it always has a disease-free equilibrium *D*_0_ = (Λ/*μ*, 0,0). Define the basic reproduction number

(4) $$\begin{array}{c} R_{0}=\frac{\Lambda \left(\left(\mu +\mu_{1}\right)\beta_{1}+\sigma \beta_{2}\right)}{\mu \left(\mu +\sigma \right)\left(\mu +\mu_{1}\right)}. \end{array}$$

Let *D*^*∗*^ = (*S*^*∗*^, *E*^*∗*^, *I*^*∗*^) be the endemic equilibrium of model (2). If *R*_0_ > 1, we can obtain a positive equilibrium *D*^*∗*^ as follows:

(5) S∗=μ+σμ+μ1μ+μ1β1+σβ2,E∗=Λμ+μ1β1+σβ2−μμ+σμ+μ1μ+σμ+μ1β1+σβ2,I∗=σΛμ+μ1β1+σβ2−μμ+σμ+μ1μ+μ1μ+σμ+μ1β1+σβ2.

**Theorem 2 .**

If *R*_0_ ≤ 1, then disease-free equilibrium *D*_0_ = (Λ/*μ*, 0,0) of model (2) is globally asymptotically stable.

**Proof**

Construct a Lyapunov function *V*(*E*, *I*) = *E* + *rI*, where *r* > 0. Obviously, *V* is nonnegative for all *t* ≥ 0 and attains zero at *D*_0_. Differentiating *V* along the trajectories of model (2), we have

(6) V˙=E˙+rI˙=β1SE+β2SI−μ+σE+rσE−rμ+μ1I.

By Theorem 1, it is obvious that *S* ≤ Λ/*μ*. If *S* = Λ/*μ*, then *E* = *I* = 0. Thus, $\dot{V}=0$. If *S* < Λ/*μ*, one has

(7) V˙<β1ΛμE+β2ΛμI−μ+σE+rσE−rμ+μ1I=β1Λμ+rσ−μ+σE+β2Λμ−rμ+μ1I.

When *R*_0_ = 1, take *r* = *β*_2_Λ/*μ*(*μ* + *μ*_1_) = (*μ*(*μ* + *σ*) − *β*_1_Λ)/*μσ*. Clearly, $\dot{V}<0$.

When *R*_0_ < 1, for

(8) $$\begin{array}{c} \frac{\beta_{2}\Lambda }{\mu \left(\mu +\mu_{1}\right)}<r<\frac{\mu \left(\mu +\sigma \right)-\beta_{1}\Lambda }{\mu \sigma }, \end{array}$$

we have $\dot{V}<0$. By the LaSalle's invariant principle, the equilibrium *D*_0_ is globally asymptotically stable.

**Theorem 3 .**

If *R*_0_ > 1, then endemic equilibrium *D*^*∗*^ = (*S*^*∗*^, *E*^*∗*^, *I*^*∗*^) of model (2) is globally asymptotically stable.

**Proof**

Construct a Lyapunov function

(9) VS,E,I=S−S∗−S∗ln⁡SS∗+E−E∗−E∗ln⁡EE∗+β2S∗I∗σE∗I−I∗−I∗ln⁡II∗.

Based on model (2), it is obvious that

(10) V˙=1−S∗SS˙+1−E∗EE˙+β2S∗I∗σE∗1−I∗II˙=1−S∗SΛ−β1SE−β2SI−μS+1−E∗Eβ1SE+β2SI−μ+σE+β2S∗I∗σE∗1−I∗IσE−μ+μ1I.

Since Λ = *μS*^*∗*^ + *β*_1_*S*^*∗*^*E*^*∗*^ + *β*_2_*S*^*∗*^*I*^*∗*^, we have

(11) V˙=−μS−S∗2S+β1S∗E∗+β2S∗I∗−β1S∗E∗S∗S−β2S∗I∗S∗S+β1S∗E+β2S∗I−μ+σE−β1SE∗−β2SIE∗E+μ+σE∗+β2S∗I∗E∗E−β2S∗I∗μ+μ1σE∗I−β2S∗I∗E∗·E·I∗I+β2S∗I∗μ+μ1σE∗I∗.

By the equations (*μ* + *μ*_1_)*I*^*∗*^ = *σE*^*∗*^, (*μ* + *σ*)*E*^*∗*^ = *β*_1_*S*^*∗*^*E*^*∗*^ + *β*_2_*S*^*∗*^*I*^*∗*^ and *R*_0_ > 1,

(12) V˙=−μS−S∗2S+2β1S∗E∗+3β2S∗I∗−β1S∗E∗S∗S−β2S∗I∗S∗S−β1S∗E∗SS∗−β2S∗I∗ISE∗I∗S∗E−β2S∗I∗EI∗E∗I=−μS−S∗2S+β1S∗E∗2−S∗S−SS∗+β2S∗I∗3−S∗S−ISE∗I∗S∗E−EI∗E∗I≤0.

By the LaSalle's invariant principle, endemic equilibrium *D*^*∗*^ is globally asymptotically stable.

## 4. Optimal Control Model

Optimal control theory is a powerful mathematical tool that can be used to a set of epidemiological models in their attempt to find the most effective control strategy to minimize the number of infected individuals [30, 31]. For example, [32] used optimal control theory to determine the condition for the elimination of tumor cells under treatment for cancer. Reference [33] studied an SIR model by using vaccination as their control. The main goal of this section is to investigate an effective strategy to control the spread of HIV/AIDS in a community.

In model (2), there are three variables *S*(*t*), *E*(*t*), and *I*(*t*). We wish that the number of *S* is in the high level and those of *E* and *I* are in the low levels. For the optimal control problem we introduce three control functions in model (2): precaution *u*_1_(*t*), screening *u*_2_(*t*), and treatment *u*_3_(*t*), which are the corresponding percentages of the variables (*S*(*t*), *E*(*t*), *I*(*t*)) per unit of time. The control function *u*_1_ is some prevention measures used by susceptible individuals to protect themselves, for instance, reducing sexual risk, alcohol and drug use, and mother-to-child risk. The control function *u*_2_ is screening of undiagnosed infected people such as sexually active gay and bisexual men and people who have injected drugs and shared needles with others. After testing, it is important to find out the result. If one is HIV positive, he (or she) should see a doctor and start HIV treatment as soon as possible. The control function *u*_3_ is the control on treatment of infected individuals.

Denote *u*(*t*) = (*u*_1_(*t*), *u*_2_(*t*), *u*_3_(*t*)). In order to minimize the number of total infected humans in *E* and *I* classes and keep total cost of precaution, screening, and treatment low during the spread, an objective function *J*(*u*) is defined by

(13) Ju=∫tstfE+I+C1u12t+C2u22t+C3u32tdt,

subject to the equations as follows:

(14) S˙t=Λ−1−u1tβ1StEt+β2StIt−μSt,E˙t=1−u1tβ1StEt+β2StIt−μ+σ+u2tEt,I˙t=σ+u2tEt−μ+μ1+u3tIt,T˙t=u3tIt−μTt,

where *T* is the number of treatment individuals. Here, *t*_*s*_, *t*_*f*_ are the start time and the final time and *C*_*i*_ is the relative cost measure of control function *u*_*i*_ for *i* = 1,2, 3. In the objective function (13), the first two terms represent benefit of *E* and *I* populations that we wish to reduce. The rest terms are benefit of minimizing the cost of control *u*. Hence, we seek an optimal control *u*^*∗*^ = (*u*_1_^*∗*^, *u*_2_^*∗*^, *u*_3_^*∗*^) such that

(15) $$\begin{array}{c} J\left(\;u^{*}\;\right)=\underset{u\in U}{\min}\left\{\;J\left(\;u\;\right):u\in U\;\right\}, \end{array}$$

where the control set *U* = {*u* = (*u*_1_, *u*_2_, *u*_3_)∣*u*_*i*_ : [*t*_*s*_, *t*_*f*_]→[0,1] are Lebesgue measurable, *i* = 1,2, 3}.

Based on Pontryagin's Maximum Principle in [34], there exists an optimal control *u*^*∗*^ with a corresponding solution (*S*_*∗*_, *E*_*∗*_, *I*_*∗*_, *T*_*∗*_) of model (14) such that *J*(*u*) is minimized over *U*. Next we provide the optimal control *u*^*∗*^ which gives the optimal levels for the various control measures and the corresponding state (*S*_*∗*_, *E*_*∗*_, *I*_*∗*_, *T*_*∗*_). In order to get necessary conditions of optimal control *u*^*∗*^, it needs to establish a Hamiltonian *H* with respect to *u*_1_, *u*_2_, and *u*_3_. Based on the cost function *J*(*u*) and model (14), the Hamiltonian is stated as

(16) H=E+I+C1u12+C2u22+C3u32+λ1Λ−1−u1β1SE−β2SI−μS+λ21−u1β1SE+β2SI−μ+σ+u2E+λ3σ+u2E−μ+μ1+u3I+λ4u3I−μT,

where *λ*_*i*_, *i* = 1,2, 3,4, are adjoint variables.

**Theorem 4 .**

Let *u*^*∗*^ ∈ *U* be an optimal control with the corresponding solutions (*S*_*∗*_, *E*_*∗*_, *I*_*∗*_, *T*_*∗*_) of model (14) such that

(17) $$\begin{array}{c} J\left(\;u^{*}\;\right)=\underset{u\in U}{\min}\left\{\;J\left(\;u\;\right):u\in U\;\right\}. \end{array}$$

Then, there exist the adjoint variables *λ*_*i*_, *i* = 1,2, 3,4, satisfying

(18) dλ1dt=1−u1β1E+β2I+μλ1−1−u1β1E+β2Iλ2,dλ2dt=−1+1−u1β1Sλ1+μ+σ+u2−1−u1β1Sλ2−σ+u2λ3,dλ3dt=−1+1−u1β2Sλ1−1−u1β2Sλ2+μ+μ1+u3λ3,dλ4dt=−μλ4

with transversality conditions *λ*_*i*_(*t*_*f*_) = 0. Moreover, the optimal control

(19) u1∗=max⁡0,min⁡λ2−λ1β1E∗+β2I∗S∗2C1,1,u2∗=max⁡0,min⁡λ2−λ3E∗2C2,1,u3∗=max⁡0,min⁡λ3−λ4I∗2C3,1.

**Proof**

Applying Pontryagin's Maximum Principle, we differentiate Hamiltonian *H* with respect to *S*, *E*, *I*, and *T*, respectively. The adjoint system can be written as

(20) dλ1dt=−∂H∂S,λ1tf=0,dλ2dt=−∂H∂E,λ2tf=0,dλ3dt=−∂H∂I,λ3tf=0,dλ4dt=−∂H∂T,λ4tf=0,

which results in the stated adjoint system (18). By considering the optimal condition ∂*H*/∂*u*_*i*_ = 0 for *i* = 1,2, 3, we get the optimal control *u*^*∗*^ subject to the constraint set *U*; the characterization (19) can be derived.

## 5. Parameter Analysis and Estimation

In model (2), there are six parameters: Λ, *β*_1_, *β*_2_, *μ*, *μ*_1_, and *σ*. Based on National Bureau of Statistics of China [35], the data of birth and natural death rates are collected from 2004 to 2016; see Table 1.

**Figure 4:**
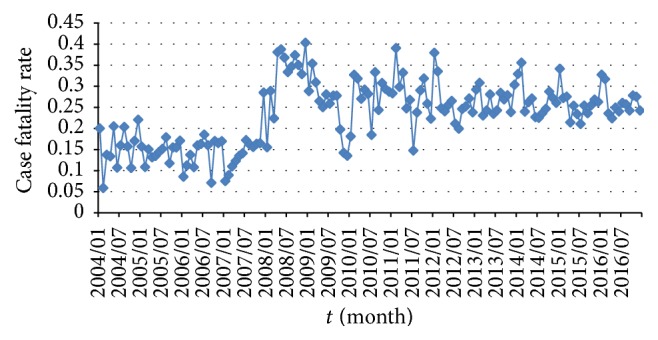
Trajectory of case fatality rate *μ*_1_ by month, 2004–2016.

The whole population of China in 2003 is 1,292,270,000. Based on the mean of birth and death rates in the last column of Table 1, the recruitment rate can be estimated by

(21) $$\begin{array}{c} \Lambda =\frac{0.01218}{12}\times \text{1,292,270,000}=\text{1,311,903}\;{month}^{-1}, \end{array}$$

and natural death rate is *μ* = 0.00698/12 = 0.00058 month^−1^. Case fatality rate *μ*_1_ is the proportion of deaths among cases per month.  Figure 4 shows that fluctuations of *μ*_1_, from 2004 to 2016, satisfy 0.05 ≤ *μ*_1_ ≤ 0.40 month^−1^.

The incubation period of HIV infected individuals ranges from 3 weeks to 20 years. Thus, transfer rate *σ* is set by constraint condition 3/4 ≤ 1/*σ* ≤ 240 months. That is, 0.0042 ≤ *σ* ≤ 1.333 month^−1^. Moreover, assume 0 < *β*_1_, *β*_2_ < 1.

On the other hand, the initial conditions *S*_0_, *E*_0_, and *I*_0_ of model (2) should be set. In model (2), *I*_0_ is the initial value of cumulative diagnosed HIV/AIDS people in January, 2004. By Figure 3, *I*_0_ = 330,839. However, it is difficult to obtain *E*_0_, which is the number of initial HIV/AIDS people who are not yet diagnosed. According to the CDC, by the end of May, 2010, more than 470,000 people with HIV have not be found or diagnosed who are unaware of their infection. Based on Figure 3, there are 382,593 alive HIV/AIDS people in May, 2010. It means that the individuals from the *E* class are larger than those of the *I* class. Since 470000/382593 = 1.2285, assume that *E*_0_ = [1.2285*I*_0_] = 406435 in this paper, where [·] is integer portion of a number. Since population number of China was 1,292,270,000 in 2003 [35], we have

(22) S0=1,292,270,000−406435−330,839=1,291,532,726.

The Nelder-Mead searching algorithm is one of the best known algorithms for multidimensional unconstrained nonlinear minimization (MUNM) [36]. We use the algorithm to find a local minimizer of the model (2) with initial conditions *S*_0_, *E*_0_, and *I*_0_ and capture the problem-dependent parameters so that the model outcome is a better fit to the real data. The algorithm starts at initial chosen values of these parameters, satisfying ranges of them in the second column of Table 2. The estimated values of parameters are shown in the last column.

**Figure 5:**
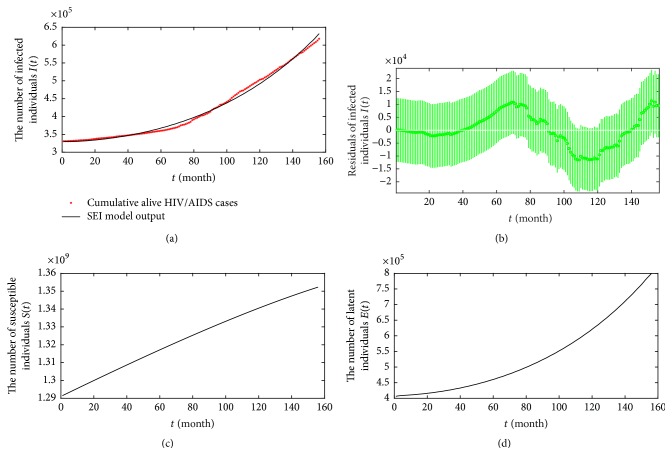
Fitting values by months from 2004 to 2016: (a) class *I*, (b) residuals analysis, (b) class *S*, and (c) class *E*.

Based on Table 2, we obtain the fitting values of class *I* and compare with cumulative alive HIV/AIDS cases from 2004 to 2016; see Figure 5(a).  Figure 5(b) is used to diagnose outliers of the fitting values. If the confidence interval of residual does not contain zero, then the residual is larger than would be expected at the 5% significance level, which is evidence that the observation is an outlier. In Figure 5(b), all confidence intervals contain zero. Thus, the residual plot demonstrates that the calculated values of the SEI model fit well the original data of class *I*. The fitting plot of class *S* is given by Figure 5(c).  Figure 5(d) shows that the population of class *E* is about 799,652 in December, 2016, which is more than that of class *I*.

By (4) and parameters in Table 2, we have *R*_0_ = 1.75 > 1. Thus, by Theorem 3, HIV/AIDS epidemic still infects humans and will be endemic in China without the effective control. There are two main reasons: (i) the number of the undiagnosed HIV/AIDS individuals is more than that of diagnosed people, and (ii) transmission rate *β*_2_ for contacting with the diagnosed HIV/AIDS patients is larger than the value *β*_1_ of class *E*. We next consider optimal control strategies on the spread of HIV/AIDS epidemic, for instance, precaution, screening, and treatment.

## 6. Optimal Control Strategies

In this section, we aim to find an optimal control strategy for the spread of HIV/AIDS epidemic in China. The optimal control is obtained by solving the control system, including objective function (13), state equations (14), adjoint equations (18), and control characterization (19). Using Runge-Kutta scheme and parameters in Table 2, we start to solve the state equations with a guess for the controls over the simulated time. Then, the state variables and initial guess are used to adjoin equations backward in time with given control characterization. This process is repeated and iterations are stopped if the values of the unknowns at the previous iterations are very close to the ones at the present iterations. Moreover, numerical simulations of process are carried out using the following values:

(i) Denote *t*_*s*_ = 0 and *t*_*f*_ = 360 months, where we analyze the optimality control system by months from January 2017 to December 2046. Based on Figure 5, for model (14) we have

(23) Sts=1352,263,692,Ets=799,652,Its=617,756,Tts=0.

(ii) Take *C*_1_ = 3 × 10^6^ RMB, *C*_2_ = 6 × 10^6^ RMB, and *C*_3_ = 2 × 10^9^ RMB, which correspond the cost of control functions *u*_1_, *u*_2_, and *u*_3_, respectively. Each control incurs some costs. Unfortunately, we do not have good data on the costs associated with these control functions. Hence, we focus on the use of “relative” cost for the control. This estimation values of *C*_1_, *C*_2_, and *C*_3_ are based on the facts that the cost *C*_1_ of precaution *u*_1_ is lower than the cost *C*_2_ of screening *u*_2_ and *C*_2_ is lower than the cost *C*_3_ of treatment *u*_3_. The cost of treatment will include the cost of drugs, medical examinations, and hospitalization.

### 6.1. Strategy A: Optimal Screening *u*_2_ and Treatment *u*_3_

**Figure 6:**
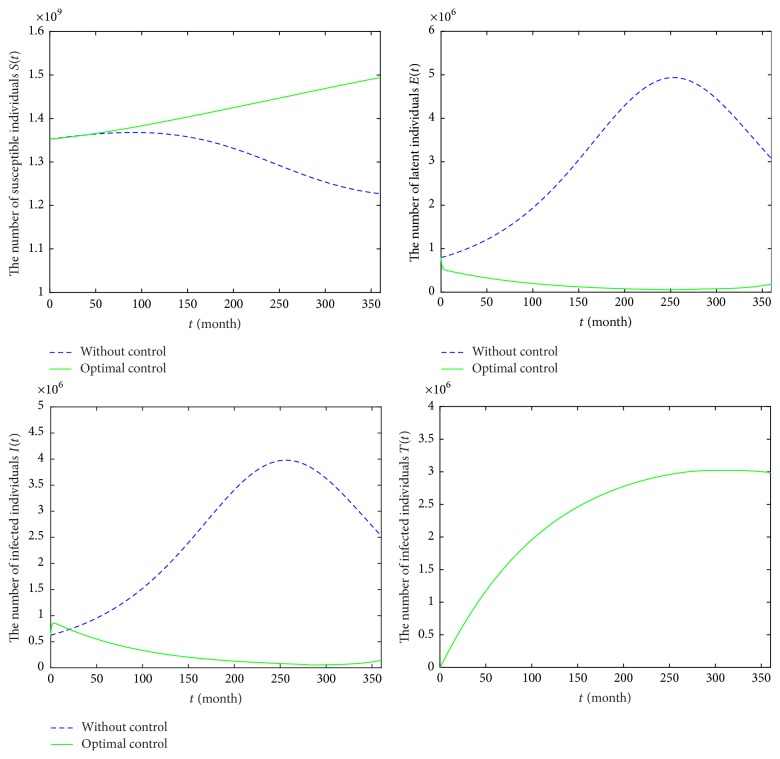
Epidemic trajectories with optimal screening *u*_2_ and treatment *u*_3_ and without optimal control during 2017–2046.

This strategy shows that the high-risk groups, such as the gay community, drug abusers, sex workers, and children of HIV positive mothers, will be screened and diagnosed. Treatment is given to the diagnosed HIV/AIDS patients. In Figure 6, the control individuals are marked by green lines while the individuals without control are marked by blue lines. Comparing Strategy A with the model (2), the number of susceptible individuals *S*(*t*) is increasing and the undiagnosed HIV patients *E*(*t*) are obviously reduced in Figure 6. However, the number of diagnosed classes *I*(*t*) firstly increases and then decreases from 2017 to 2046. The number of treatment classes *T*(*t*) increases and reaches 2,987,597 in December 2046. Obviously, the number of undiagnosed HIV/AIDS individuals is lesser when the control strategy is utilized than when the control strategy is not implemented. For the diagnosed HIV/AIDS cases with optimal control, it will be lesser than those without control strategy.

**Figure 7:**
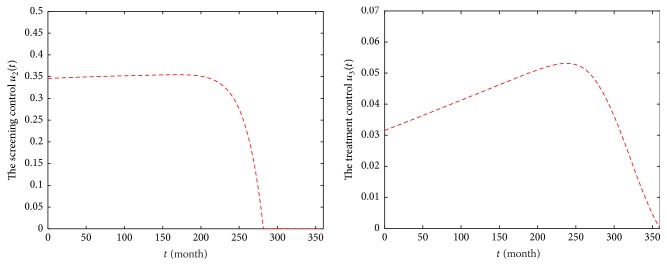
Profiles of control functions *u*_2_ and *u*_3_ in Strategy A during 2017–2046.

The control profiles are shown in Figure 7. It shows that the optimal control variables *u*_2_ and *u*_3_ at a time *t* play an important role in minimizing the infected population spreading HIV virus in the host population. The screening is administered in 0.35 nearly up to 220 months and then is tapered off, while the optimal treatment control *u*_3_ increases firstly and gradually reduces to the lower bound. This means that prevention with screening and treatment is important while the disease prevails, comparing with the results of model (2).

### 6.2. Strategy B: Optimal Precaution *u*_1_ and Treatment *u*_3_

**Figure 8:**
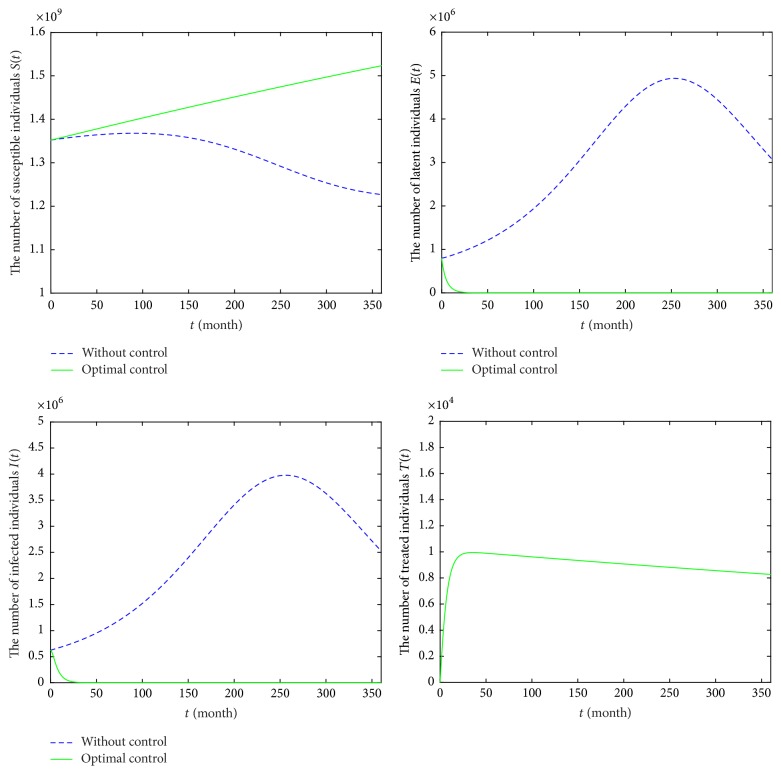
Epidemic trajectories with optimal precaution *u*_1_ and treatment *u*_3_ and without optimal control during 2017–2046.

**Figure 9:**
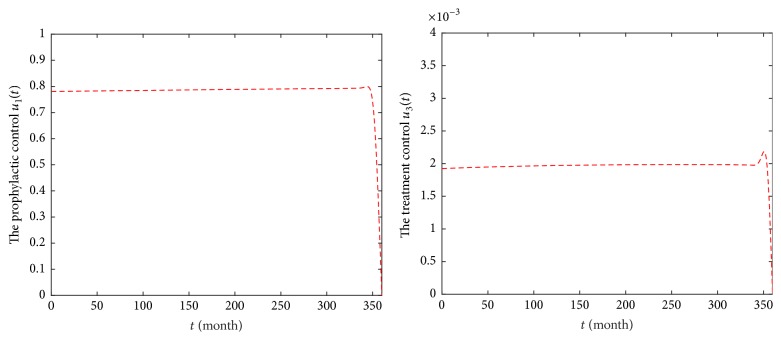
Profiles of control functions *u*_1_ and *u*_3_ in Strategy B during 2017–2046.

For Strategy B, it reflects that precaution is optimal for susceptible individuals and treatment is given to the diagnosed HIV/AIDS patients. In Figure 8, it is observed that the number of classes *S*(*t*) in model (14) is increasing, comparing with that of model (2). The numbers of classes *E* and *I* are obviously decreasing in model (14) and they will reach the lower values after 83 months. Moreover, the number of classes *S*(*t*) is 1,523,291,482, and population of *E* class (or *I* class) will reach zero in December 2046. The number of treatment classes *T*(*t*) increases for 36 months and then decreases by 8,270. Obviously, Strategy B is better than Strategy A.  Figure 9 provides the profiles of control functions *u*_1_ and *u*_3_. Obviously, there are a constant precaution and a constant treatment required for nearly the entire length of the strategy.

### 6.3. Strategy C: Optimal Precaution *u*_1_, Screening *u*_2_, and Treatment *u*_3_

**Figure 10:**
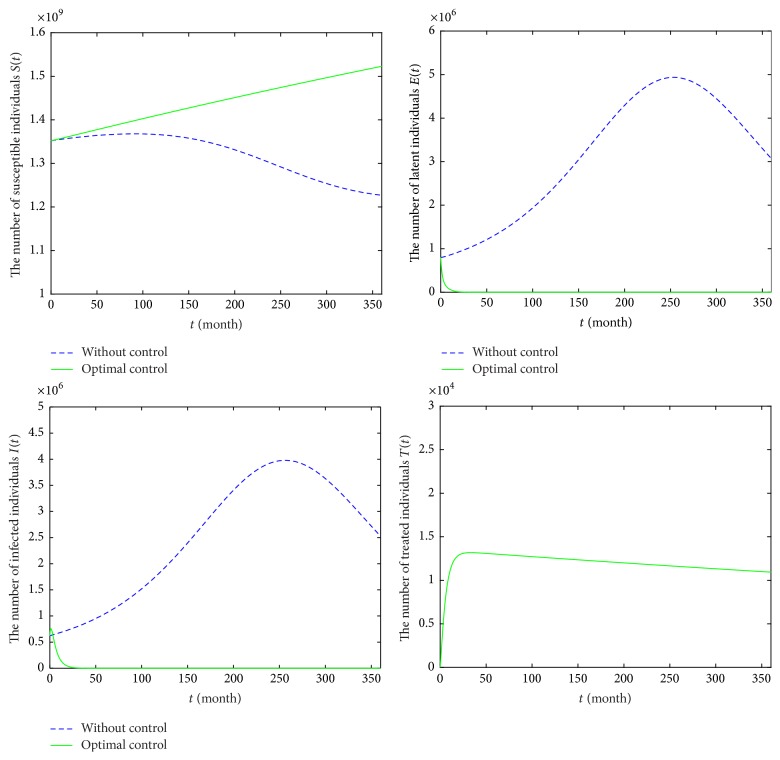
Epidemic trajectories with optimal precaution *u*_1_, screening *u*_2_, and treatment *u*_3_ and without optimal control during 2017–2046.

**Figure 11:**
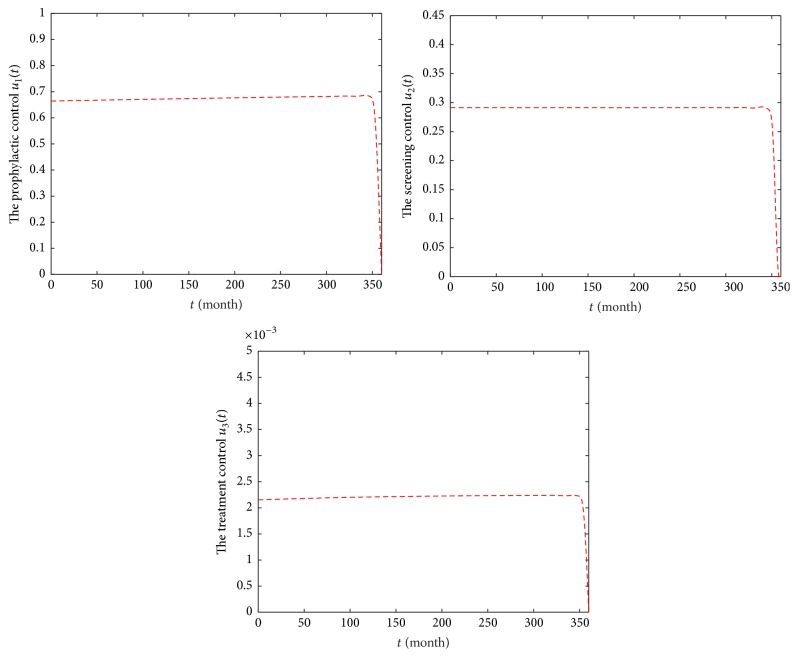
Profiles of control functions *u*_1_, *u*_2_, and *u*_3_ in Strategy C during 2017–2046.

Strategy C means that the precaution, screening, and treatment are important in HIV/AIDS epidemic.  Figure 10 shows that, under Strategy C, people of class *S* reach 1,523,013,518, and population of *E* class (or *I* class) will reach zero in December 2046, similar to those of Strategy B. The number of classes *T* increases for 33 months and then decreases by 10,937, which is larger than that of Strategy B. In Figure 11, we observe that there are a constant precaution, a constant screening, and a constant treatment, required for nearly the entire length of the strategy.

Comparing the results of model (2) and Strategies A, B, and C, Table 3 shows that HIV/AIDS epidemic will be endemic under model (2) and Strategy A and will be extinct in China under strategies B and C. Our conclusion from this is intervention practices that involve both precaution and treatment controls yield a relatively better result.

## 7. Discussion and Conclusion

More and more susceptible individuals are at risk of HIV infection in China since 2004. In order to analyze the spread of HIV/AIDS epidemic, we first collect the available HIV/AIDS data. In general, we can obtain the numbers of new HIV/AIDS cases and disease-caused death cases by NHFPC, CDC, and NBS of China. However, people are mainly infected by the alive diagnosed and undiagnosed people with HIV, through kinds of transmission routes. By calculating, we provide the numbers of cumulative alive HIV/AIDS and cumulative disease-caused death cases from January 2004 to December 2016.

Based on the transmission of HIV/AIDS and the corresponding data, conclusions may be summarized as follows:

- A modified SEI model (2) is established. By Lyapunov functions, the globally asymptotical stabilities of two equilibria are discussed. Among all parameters of the model, recruitment rate Λ and natural death rate *μ* are given by local demography. Other parameters such as contact rates *β*_1_, *β*_2_, transfer rate *σ*, and case fatality rate *μ*_1_ are estimated by MUNM method. By the definition of *R*_0_, the result shows that HIV/AIDS epidemic is prevalent in China since *R*_0_ > 1.
- In order to find an optimal control strategy to control the spread of HIV/AIDS epidemic in China, an objective function is introduced, satisfying the SEI model (2) with control functions. Characterization of optimal controls is analyzed. Based on some results of model (2), we use Runge-Kutta method to analyze optimal control of HIV epidemic from 2017 to 2046 and provide three strategies: A, B, and C. The results show Strategy B is a better choice than Strategies A and C and model (2) without control. That is, the precaution and treatment are two effective approaches to prevent and control the spread of HIV/AIDS epidemic.

Each country has its own HIV/AIDS data. Here, we focus on China, but the same analysis can be applied to other countries.

## Figures and Tables

**Table 1:** Birth and death rates in China from 2004 to 2016 (year^−1^).

Year														
	2004	2005	2006	2007	2008	2009	2010	2011	2012	2013	2014	2015	2016	Mean
Birth rate (‰)	12.29	12.4	12.09	12.1	12.14	11.95	11.9	11.93	12.1	12.08	12.37	12.07	12.95	12.18
Natural death rate (‰)	6.42	6.51	6.81	6.93	7.06	7.08	7.11	7.14	7.15	7.16	7.16	7.11	7.09	6.98

**Table 2:** Estimated values of model parameters.

Parameter	Definition (unit: per month^−1^)	Range	Initial value	Estimated value
*β*_1_	The transmission rate of the latent infection individuals	(0,1]	1.25 × 10^−13^	1.05 × 10^−12^
*β*_2_	The transmission rate of the infected individuals	(0,1]	1.7 × 10^−10^	2.7 × 10^−10^
*σ*	The immigration rate of the latent infection individuals	[0.004,1.333]	0.5	0.28
*μ*_1_	Case fatality rate	[0.05,0.40]	0.065	0.35

**Table 3:** Comparison among model (2) and control strategies A, B, and C in December, 2046.

Control	*S*	*E*	*I*	*T*
Without control	1226879802	3067415	2527663	—
Control strategy A	1494098461	186888	140873	2987597
Control strategy B	1523291482	0	0	8270
Control strategy C	1523013518	0	0	10937
